# A method to generate perfusable physiologic-like vascular channels within a liver-on-chip model

**DOI:** 10.1063/5.0170606

**Published:** 2023-12-04

**Authors:** E. Ferrari, E. Monti, C. Cerutti, R. Visone, P. Occhetta, L. G. Griffith, M. Rasponi

**Affiliations:** 1 Department of Electronics, Information and Bioengineering, Politecnico di Milano, via Camillo Golgi 39, 20134 Milano (MI), Italy; 2 BiomimX Srl, viale Decumano 41, 20157 Milano (MI), Italy; 3 Istituto Europeo di Oncologia, via Adamello 16, 20139 Milano (MI), Italy; 4 Department of Biological Engineering, Massachusetts Institute of Technology, 77 Massachusetts Ave., Cambridge, Massachusetts 02139, USA

## Abstract

The human vasculature is essential in organs and tissues for the transport of nutrients, metabolic waste products, and the maintenance of homeostasis. The integration of vessels in *in vitro* organs-on-chip may, therefore, improve the similarity to the native organ microenvironment, ensuring proper physiological functions and reducing the gap between experimental research and clinical outcomes. This gap is particularly evident in drug testing and the use of vascularized models may provide more realistic insights into human responses to drugs in the pre-clinical phases of the drug development pipeline. In this context, different vascularized liver models have been developed to recapitulate the architecture of the hepatic sinusoid, exploiting either porous membranes or bioprinting techniques. In this work, we developed a method to generate perfusable vascular channels with a circular cross section within organs-on-chip without any interposing material between the parenchyma and the surrounding environment. Through this technique, vascularized liver sinusoid-on-chip systems with and without the inclusion of the space of Disse were designed and developed. The recapitulation of the Disse layer, therefore, a gap between hepatocytes and endothelial cells physiologically present in the native liver milieu, seems to enhance hepatic functionality (e.g., albumin production) compared to when hepatocytes are in close contact with endothelial cells. These findings pave the way to numerous further uses of microfluidic technologies coupled with vascularized tissue models (e.g., immune system perfusion) as well as the integration within multiorgan-on-chip settings.

## I. INTRODUCTION

The human vascular system is responsible for essential biological functions in organs and tissues, such as the transport of nutrients, signaling molecules, metabolic waste products, and circulating cells, as well as the maintenance of environmental variables (e.g., temperature and pH) and homeostasis.^1,2^ Also, drug administration into the human body takes place by means of the vascular system. Thus, to develop physiologically relevant models for drug toxicity and safety testing, the inclusion of engineered vasculature has been considered in the most recently developed *in vitro* biomimetic systems.^3–8^

Among all, the liver is involved in the metabolism of drugs and xenobiotics whose metabolism depends on the hepatic physiology, which is closely correlated with tissue architecture. The liver minimal functional unit is the hepatic sinusoid, a specialized capillary of fenestrated liver sinusoidal endothelial cells (LSECs) separated from hepatocytes by an extracellular matrix (ECM) proteic layer named the space of Disse.^9^ LSECs not only constitute a physical barrier but also have an active role in regulating the exchange of nutrients and the functional maintenance of hepatocytes through paracrine actions.^10^ The recapitulation of the three-dimensional (3D) hepatic architecture of the sinusoid, comprising the crosstalk between LSECs and hepatocytes, is paramount for the generation of a functional *in vitro* hepatic model suitable for the pre-clinical phases of the drug development pipeline (DDP). In recent years, different strategies have been exploited to recreate an *in vivo*-like hepatic microenvironment including vasculature. Some models exploit either porous membranes^5^ or scaffolds^6^ to support the culture of endothelial cells, physically separating them from the hepatocytes. However, similar physical barriers are not present in the native hepatic *milieu*. Moreover, these models fail to recapitulate the intrinsic circularity of vascular channels, which is known to promote barrier functionality and cytoskeletal alignment of endothelial cells.^11^ Some attempts have been conducted with sacrificial bioprinting technique^7^ or cast-molding (e.g., sacrificial needles)^8^ to create vascularized hollow microchannels within hepatocytes-laden hydrogels. However, such attempts did not take into consideration the space of Disse. In this work, we developed a method to generate perfusable vascular channels within 3D hepatic microtissues, without the need to introduce any artificial physical separation between hepatocytes and endothelial cells. To this aim, we designed two liver-on-chip (LoC) platforms, with and without the inclusion of an ECM layer, mimicking the space of Disse. In particular, the platform developed to recapitulate the space of Disse, named ECM device, principally differs from the other one, named DC device, from a 100 *μ*m-thick collagen–fibrin layer interposed between hepatocytes and endothelial cells. In particular, the platforms allow the generation of a vascularized circular channel of 120–140 *μ*m diameter inside a low-volume chamber hosting a primary human hepatocytes (PHH)-based 3D hepatic construct. The hepatic functionality (e.g., albumin production and CYP3A4 activity) was assessed under the influence of both perfusion, with endothelial cells experiencing *in vivo*-like shear stress,^9^ and the presence of the space of Disse. Furthermore, the presented method was demonstrated to be amenable for integration within multiorgan-on-chip systems (see supplementary material S.4), where recapitulating liver functionality and metabolism is of fundamental importance for drug screening and safety applications, as we recently showed with a liver–heart device.^12^

## II. RESULTS AND DISCUSSION

### A. Device fabrication and characterization

**FIG. 1.**
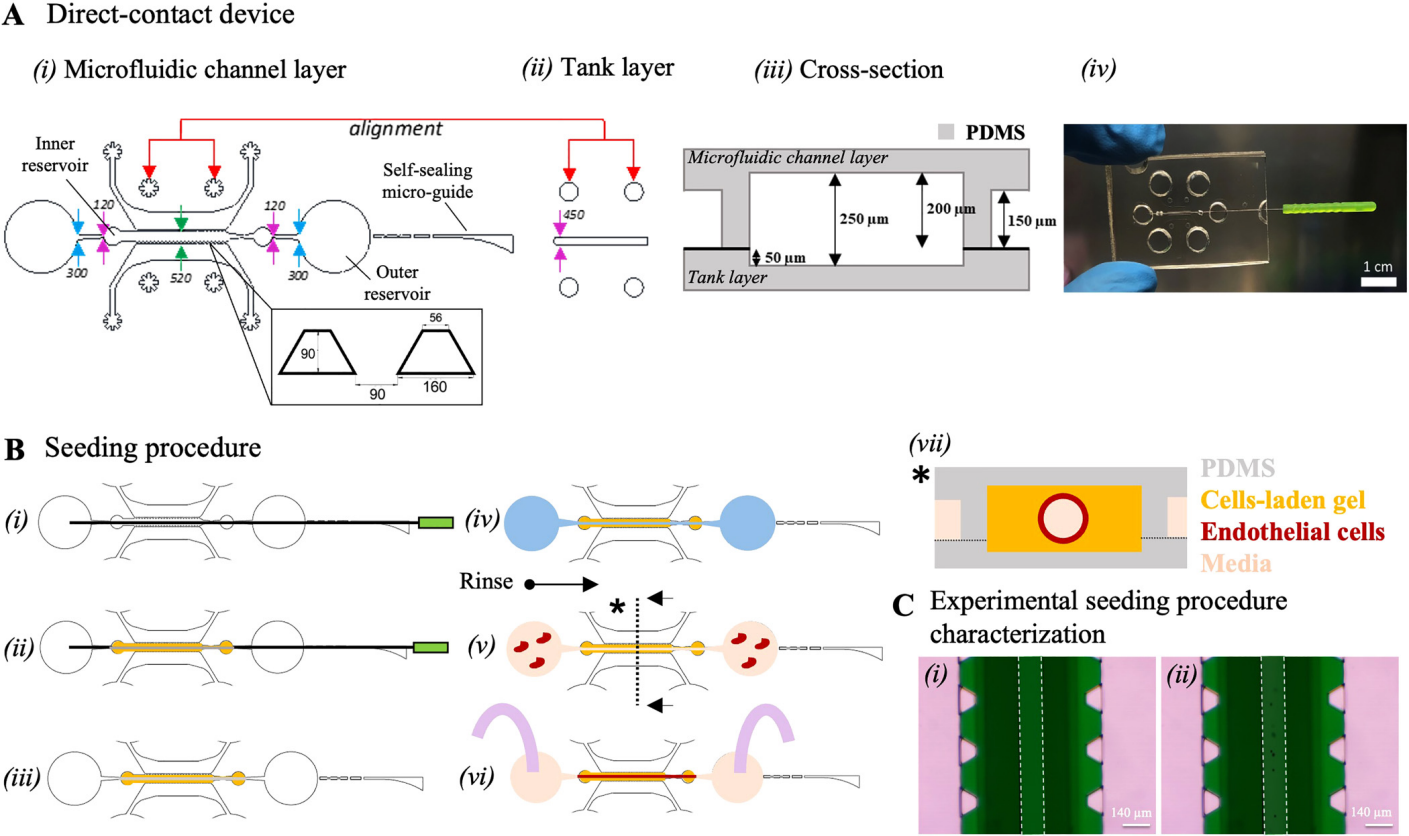
(a) Design of the direct-contact device. (i) The microfluidic channel layer is composed of one central channel separated from two media channels by two arrays of trapezoidal microposts. The layer comprises one self-sealing micro-guide to facilitate the manual insertion of a micro-needle. (ii) The tank layer is composed of a central channel to be aligned to the central channel of the microfluidic channel layer. (iii) Cross section with dimensions of the direct-contact platform. (iv) Fabricated DC platform. Scale bar is 1 cm. (b) General seeding procedure of the direct-contact device: (i) a stainless-steel needle is inserted through the guide of the device; (ii) a cell-laden gel is injected through the central seeding channel containing the needle; after polymerization, (iii) the needle is removed and (iv) fibronectin solution is injected to coat the lumen. Fibronectin is then removed and, after rinsing with medium, (v) endothelial cells are seeded within the lumen. Endothelial cells attach to the walls of the internal lumen to generate a cylindrical vascular channel that can then be (vi) perfused. (vii) Cross-sectional area of the direct-contact device, once all the passages to obtain the complete LoC model have been performed. (c) Characterization of the lumen through beads perfusion: (i) cylindrical channel formed with the procedure described in (b) and (ii) injection of polystyrene microbeads to verify lumen formation. Scale bars are 140 *μ*m.

At first, a device allowing the generation of a vascular channel embedded in cell-laden ECM-like environment was designed and fabricated. Such a device, named Direct-contact (DC) platform, was conceived as the assembly of two vertically aligned layers: (i) a microfluidic channel layer and (ii) a tank layer. The microfluidic channel layer is composed of one central channel [520 *μ*m wide, Fig. 1(a)(i), green arrows] separated from two flanking media channels by two arrays of trapezoidal microposts. Additionally, the microfluidic channel layer displays one self-sealing micro-guide featuring a squared cross section that was designed with a widening of about 1 mm at the outer border to facilitate the manual insertion of a micro-needle. The micro-guide was separated from the central channel by a 200 *μ*m pierceable and self-sealing wall.^13^ Moreover, the central culture channel contains a couple of inner and outer reservoirs, for cell-laden hydrogel injection and endothelial cell seeding, respectively. The inner reservoir is connected to the outer one by a nozzle and a diverging channel enabling cell seeding and avoiding leakages outside the inner reservoir. The diverging channel has the smaller width of 120 *μ*m (in contact with the inner reservoir, pink arrows) and the larger width of 300 *μ*m (in contact to the outer reservoir, light-blue arrows) [Fig. 1(a)(i)]. The tank layer is composed of a unique 450 *μ*m wide central chamber [Fig. 1(a)(ii), pink arrows]. During the fabrication procedure, the two layers are precisely aligned to generate a total height of the central channel of 250 *μ*m [Fig. 1(a)(iii)]. Figure 1(a)(iv) shows a picture of the fabricated DC device where the needle location can be appreciated.

Once the DC device is assembled, subsequent steps are needed to obtain the complete LoC model [Fig. 1(b)]: (i) a 120 *μ*m diameter needle previously coated with Bovine Serum Albumin (BSA) is inserted through the guide until it reaches the opposite outer port; (ii) a cell-laden gel is injected through the central seeding channel containing the needle; after polymerization (iii) the needle is carefully removed leaving behind a hollow lumen inside the central gel channel. At this point, (iv) fibronectin solution is injected through the outer seeding ports to coat the lumen; the lumen is then rinsed with a medium before endothelial cells seeding (v). Endothelial cells attach to the inner wall of the lumen to generate a cylindrical vascular channel (vi) that can finally be perfused. Figure 1(b)(vii) shows the end-point concept of the cross-sectional area of the DC device.

Before biological experiments, the DC device was first characterized by means of bead perfusion within the central channel. A mix of fibrin and collagen solution was injected into the central channel [Fig. 1(c)(i)], following the steps previously described. To verify lumen formation after needle removal, a solution of polystyrene microbeads was injected through either of the outer seeding ports [Fig. 1(c)(ii)]. The complete absence of any bead in the surrounding gel confirmed the mechanical stability of the generated lumen, further excluding any microscopic leakage.

### B. Endothelial channel generation

**FIG. 2.**
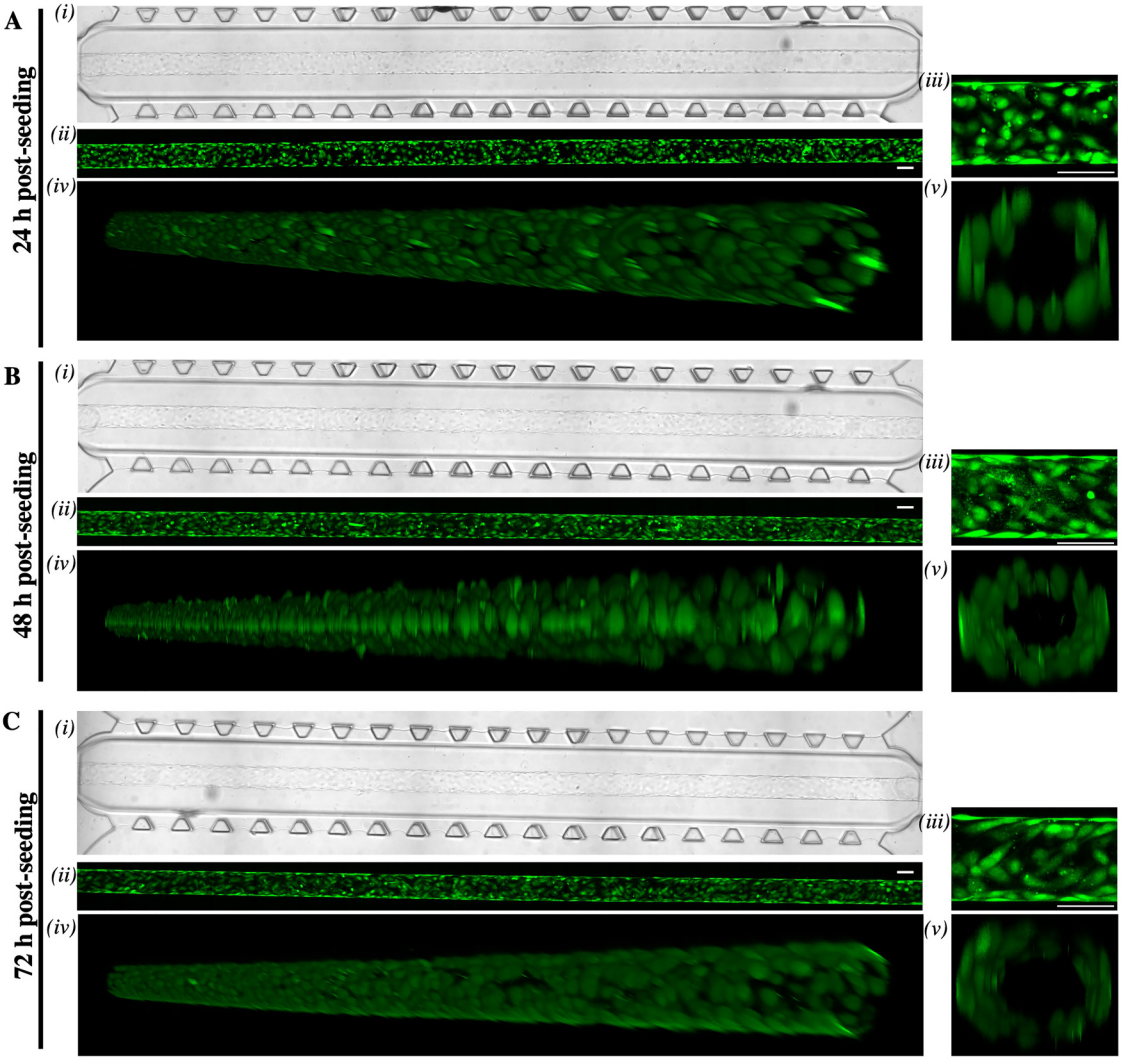
HUVEC cylindrical cell monolayer generation. Time-course of fluorescently labeled HUVEC growth to form a cylindrical monolayer at (a) 24 h (b) 48 h, and (c) 72 h post-seeding. (i) Bright field and (ii) z-stacks confocal maximal projection (25×/0.95 water immersion objective) tile-scan acquisitions of the central lumen within the DC device, full length. (iii) Enlargements of (ii), z-stacks confocal maximal projection of a single tile of the central lumen within the DC device. DC device. (iv) Central lumen full length tile-scan z-stacks and (v) y-stack central lumen cross section confocal 3D renderings. Scale bar (white), 100 *μ*m.

The generation of an endothelial monolayer in the lumen was assessed through human umbilical vein endothelial cells expressing GFP (GFP-HUVEC). Confocal images acquired during static culture time show the growth of GFP-HUVEC into a cylindrical monolayer within the DC platform at 24 h [Fig. 2(a)], 48 h [Fig. 2(b)], and 72 h [Fig. 2(c)] after seeding. It is worth noticing that the presence of the lower tank layer allowed the formation of a circular lumen completely surrounded by a gel matrix layer (i.e., 70% collagen–30% fibrin), with a thickness of at least 50 *μ*m, which is suitable to accommodate cells. The GFP-HUVEC layer maintained its circularity within the central channel of the microfluidic platform for at least one week of the culture and expressed peculiar endothelial markers such as ZO-1, VE-Cadherin, and phalloidin (see supplementary material S.2), making it amenable for the implementation within the complete LoC model.

### C. PHH viability and functionality assessment

**FIG. 3.**
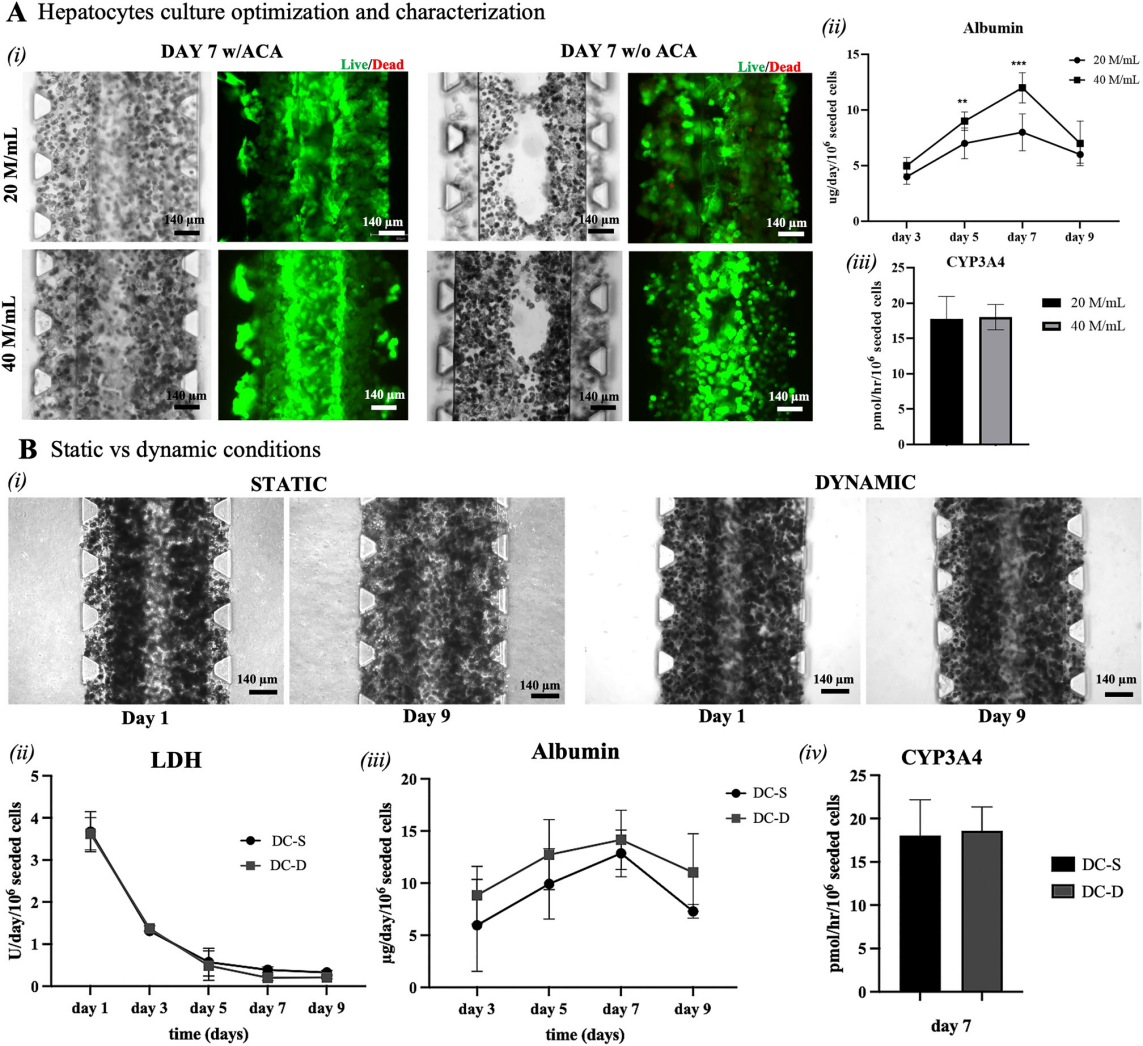
(a) Optimization of hepatocytes seeding density and culture medium. (i) Morphological and viability comparison between 20 and 40 M/ml seeding densities with a medium supplemented with ACA or without it. In the absence of ACA, the hepatocytes remodel the gel by impairing the circularity of the central lumen. Scale bars are 140 *μ*m. (ii) Hepatocytes secrete higher albumin levels when seeded at 40 M/ml while (iii) CYP3A4 activity is similar between the two seeding densities. Statistical analysis was performed using analysis of variance (ANOVA) with the Bonferroni correction: ** p = 0.002, *** p < 0.001 (N = 3). (b) Comparison between static (DC-S) and dynamic (DC-D) conditions applied to the platform. (i) Pictures on day 1 (24 h after hepatocytes seeding) and day 9 (end of the culture). Hepatocytes maintain their characteristic morphology throughout the culture without any observable differences between the conditions. Scale bars are 140 *μ*m. (ii) LDH release decreased from day 3 indicating high cellular viability. (iii) Hepatocytes produce higher levels of albumin in the dynamic condition (DC-D) compared to static condition (DC-S). (iv) CYP3A4 levels are similar between the two conditions.

The present study aims at developing a device able to recapitulate the intrinsic functionality of the liver sinusoid. First, primary human hepatocytes (PHHs) were adopted and embedded in a mix of 70% collagen (9 mg/ml) and 30% fibrin (2.1 mg/ml) gel,^14^ and their functionality at different seeding densities within the DC platform was evaluated. Such a hydrogel was indeed chosen as an initial hepatic ECM-like composition but as previously reported,^14^ it would likely evolve during culture time as additional protein deposition may occur. Notably, the adopted hydrogel exhibits physiologic liver stiffness values (see supplementary material S.1).^15,16^ The effect of medium supplemented with 2 *μ*g/ml aminocaproic acid (ACA) was assessed on PHH seeded at either low (20 M/ml) or high (40 M/ml) cell density. As shown in Fig. 3(a)(i), when ACA was supplemented, the hepatic construct could maintain its environmental organization for one week of the culture, whereas when ACA was not added, PHH remodeled the gel, and the central lumen could not be maintained in either seeding density condition. Additionally, ACA did not impair cell viability as in all conditions PHH maintained a high viability of ∼98%. ACA was, thus, adopted for the subsequent experiments.

For what concerns, hepatic functionality, human albumin concentration, and CYP3A4 activity were measured. Indeed, albumin is the most abundant plasma protein that circulates in the bloodstream, and it is produced only by the hepatocytes; a low serum albumin indicates poor liver cell function.^17^ CYP3A4 is an enzyme of the CYP450 family that participates in the biotransformation of over 50% of commonly used drugs;^18^ similarly, a low CYP activity indicates poor metabolic functions. In the DC platform, PHH maintained higher albumin levels during the entire culture period when seeded at higher concentrations, with statistically higher values on day 5 and day 7 [Fig. 3(a)(ii)]. In detail, in the 20 M/ml condition, albumin levels were 4.08 ± 0.66 (day 3), 6.98 ± 1.38 (day 5), 8.46 ± 1.65 (day 7), 6.01 ± 0.76 (day 9) *μ*g/day/106 seeded cells, whereas in the 40 M/ml albumin levels were 5.12 ± 0.75 (day 3), 9.2 ± 0.83 (day 5), 11.89 ± 1.36 (day 7), 7.33 ± 2 (day 9) *μ*g/day/10^6^ seeded cells. Regarding CYP3A4 activity on day 7 of the culture, in the 20 M/ml condition, PHH expressed 17.8 ± 3.14 pmol/h/10^6^ seeded cells, whereas in the 40 M/ml condition, they expressed 18.02 ± 1.77 pmol/h/10^6^ seeded cells with no statistical significance [Fig. 3(a)(iii)]. Based on these results, the condition 40 M/ml with ACA-supplemented medium was adopted for the subsequent experiments.

It is well known that dynamic culture conditions (i.e., medium flowing in the devices that generates a shear stress on the cells) improve cell alignment and functionality.^19^ To assess whether a dynamic medium flow could enhance PHH functionality in the DC platform, a bidirectional flow was introduced through the vascular channel by means of a rocker platform (see supplementary material S.3), controlled to apply an oscillatory shear stress of 0.9 dyne/cm^2^, comparable with that observed in the liver sinusoid *in vivo*.^20^ Figure 3(b)(i) shows that PHH maintained their characteristic morphology in both static and dynamic conditions for up to 9 days of the culture, with no significant differences. Cell viability was monitored for the whole culture time by analyzing the production of lactate dehydrogenase (LDH). Figure 3(b)(ii) shows that on day 1 of the culture, there was a LDH release of 3.6 ± 0.4 U/day/10^6^ seeded cells in both culture conditions. LDH release is a sign of cell mortality and may be due to the hepatocyte seeding process itself as well as the needle removal. On day 3, lDH release decreased to 1.31 ± 0.06 and 1.37 ± 0.1 for the direct-contact-static (DC-S) condition and the direct-contact-dynamic (DC-D) condition, respectively, further decreasing to 0.33 ± 0.05 and 0.21 ± 0.03 on day 9 for DC-S and DC-D, respectively, with no statistical difference between culture conditions. Considering albumin production, in the dynamic conditions PHH produced higher levels of albumin compared to static conditions [Fig. 3(b)(iii)]. Albumin levels were 5.95 ± 4.42, 9.92 ± 3.37, 12.85 ± 2.24, 7.3 ± 0.66 *μ*g/day/10^6^ seeded cells for the DC-S and 8.84 ± 2.77, 12.73 ± 3.36, 14.15 ± 2.84, 11.02 ± 3.72 *μ*g/day/10^6^ seeded cells for the DC-D, on days 3, 5, 7, and 9, respectively. Albumin production trend was similar among all conditions, with the highest production on day 7 of the culture; however, with no statistical significance. CYP3A4 activity on day 7 of the culture was found to be similar between static and dynamic conditions, with no statistical differences [Fig. 3(b)(iv)]. In particular, CYP3A4 levels were 18.03 ± 4.14 and 18.6 ± 2.74 pmol/h/10^6^ seeded cells for the DC-S and DC-D conditions, respectively.

Overall, the presence of flow in the DC device seems to slightly enhance liver cell functionality, however, with no statistical significance compared to static conditions. This positive trend may be due to the application of physiological shear stress on the cells as well as in an improvement of nutrients and waste product exchange. In particular, CYP3A4 activity is much higher compared to the one of freshly isolated hepatocytes,^4^ making the model amenable for *in vitro* drug testing applications.

### D. Development and validation of vascularized hepatic models

**FIG. 4.**
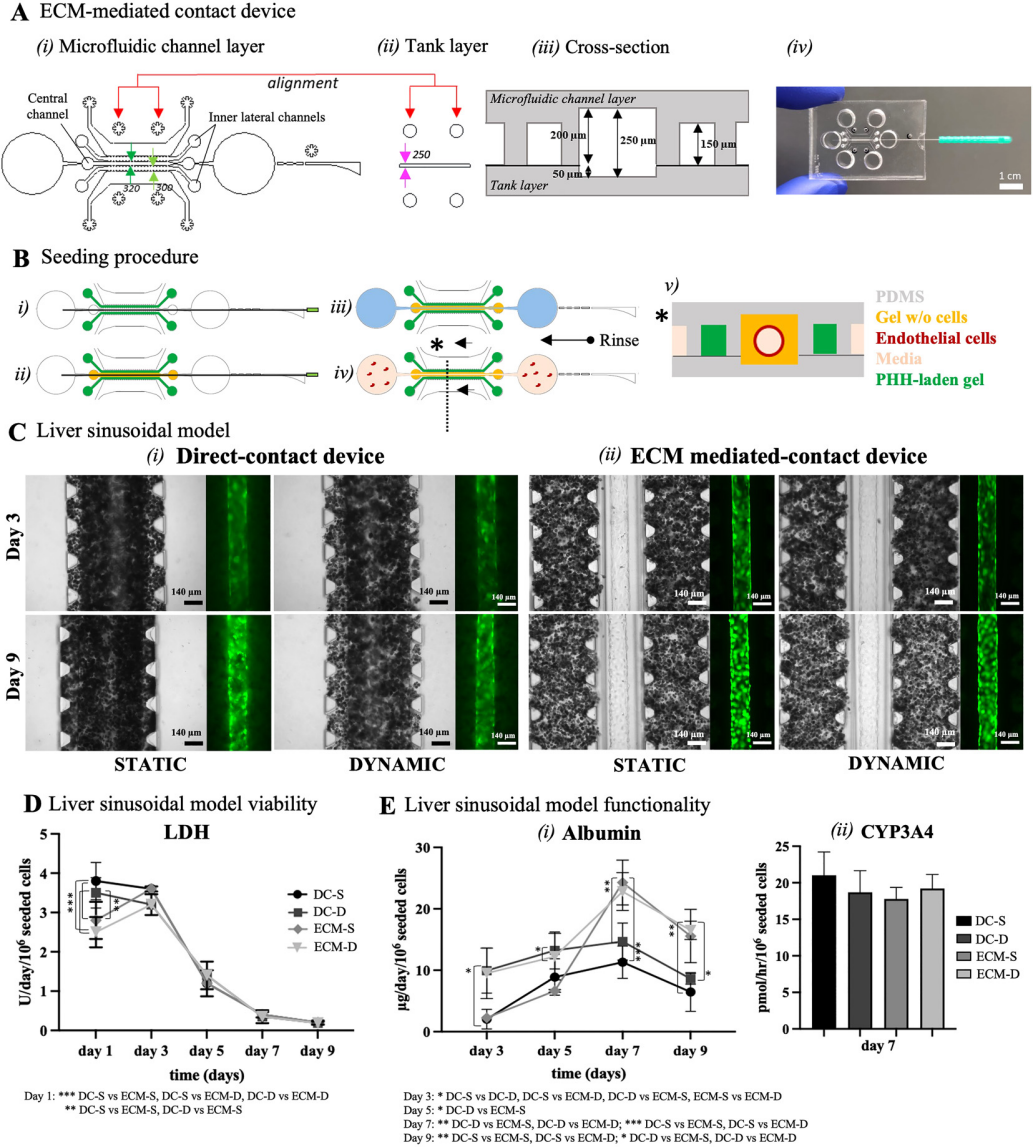
(a) Design of the ECM-mediated-contact device. (i) Microfluidic channel layer is composed of one central channel, two adjacent culture channels, and two media channels. Every channel is separated from the adjacent one by two arrays of trapezoidal microposts. The layer comprises one self-sealing micro-guide to facilitate the manual insertion of a micro-needle. (ii) The tank layer is composed of a central channel to be aligned to the central channel of the microfluidic channel layer. (iii) Cross section with dimensions of the ECM-mediated-contact platform. (iv) Fabricated ECM device. Scale bar is 1 cm. (b) Seeding procedure of the ECM-mediated-contact device: (i) PHH-laden collagen–fibrin gel is injected through the inner lateral channels; after polymerization, (ii) collagen–fibrin gel is injected in the central channel containing the needle; (iii) the needle is removed, and the fibronectin coating (in light blue) is performed; then, after rinsing with the medium, (iv) endothelial cells are seeded within the lumen to generate a cylindrical vascular channel. (v) Cross-sectional area of the ECM-mediated-contact device, once all the passages to obtain the model have been performed. (c) Liver sinusoidal model generated with PHH and GFP-HUVEC within the (i) direct-contact device and the (ii) ECM-mediated-contact device. Pictures on day 3 (24 h after GFP-HUVEC seeding) and day 9 (end of the experiment) of the culture under static and dynamic conditions. Scale bars are 140 *μ*m. (d) LDH release starts to decrease from day 5 in all conditions, indicating high cellular viability. Statistical analysis was performed using two-way ANOVA with the Tukey correction: ** p = 0.002, *** p < 0.001. ECM-S, ECM-mediated-contact device in static condition; ECM-D, ECM-mediated-contact device in dynamic condition. (e) High cellular viability is confirmed by albumin production and CYP3A4 activity levels. (i) Albumin production is higher in the ECM-mediated-contact device, as compared to the direct-contact device. The highest albumin production occurs on day 7 of the culture, and the production is higher in the ECM-mediated-contact device (static 24.29 ± 3.65 and dynamic 22.82 ± 3.08 *μ*g/day/million cells) compared with the direct-contact device (static 11.31 ± 2.63 and dynamic 14.68 ± 3 *μ*g/day/million cells). Statistical analysis was performed using two-way ANOVA with the Tukey correction: * p = 0.033 ** p = 0.002, *** p < 0.001. (ii) CYP3A4 levels are high in all conditions in both devices.

The native liver sinusoid comprises the space of Disse, an ECM layer separating the hepatocytes from the liver sinusoidal endothelial cells. With the aim to replicate such space of Disse, the ECM-mediated-contact platform (ECM) was developed, to better mimic the *in vivo* microenvironment. The ECM platform maintains the two-layer structure of the previously described DC platform. However, the microfluidic channel of the ECM layer comprises two inner lateral channels (300 *μ*m wide, light-green arrows) interposed between the central channel (320 *μ*m wide, green arrows) and the two flanking media channels [Fig. 4(a)(i)]. The tank layer is composed of a unique 250 *μ*m wide central chamber [Fig. 4(a)(ii), pink arrows]. All channels are separated from the adjacent ones by arrays of trapezoidal microposts. Figure 4(a)(iv) shows a picture of the assembled ECM device where the needle location can be appreciated.

Similar to the DC platform, once the ECM device is precisely assembled, subsequent steps are needed to obtain the complete LoC model [Fig. 4(b)]: (i) a 120 *μ*m diameter needle previously coated with BSA is inserted through the guide until it reaches the opposite outer port and PHH-laden collagen–fibrin gel is injected through the midseeding channels; after polymerization (ii) a bare collagen–fibrin gel is injected through the central seeding channel containing the needle; after polymerization (iii) the needle is carefully removed leaving behind a hollow lumen inside the central channel gel that can be coated with fibronectin; then (iv) fibronectin is removed, the lumen is rinsed with medium and endothelial cells can be seeded and allowed to adhere to the inner walls of the lumen to generate a cylindrical vascular channel. Figure 4(b)(v) shows the concept of the cross-sectional area of the ECM-mediated-contact device.

To compare the influence of the space of Disse, human umbilical vein endothelial cells (HUVECs) were used to generate a vascularized lumen within PHH-laden collagen–fibrin gel in both DC and ECM platforms. Both platforms were cultured either in static or dynamic (i.e., rocker) conditions. Endothelial cells were able to generate a circular endothelial channel within the devices well maintained for up to 9 days of the culture in both static and dynamic conditions, with no differences in terms of cell morphology [Fig. 4(c)].

On day 1 of the culture, the LDH release was of 2.6 ± 0.4 and 3.7 ± 0.5 U/day/10^6^ seeded cells in ECM and DC conditions, respectively [Fig. 4(d)]. This is probably due to the PHH seeding process. In fact, we see a higher LDH release in the DC platform compared to the ECM one probably because the needle is in direct ,contact with PHH and its removal can detach the surrounding cells, thus increasing cell mortality in the model. Similar levels of LDH were measured also on day 3 of the culture: specifically, 3.6 ± 0.5 U/day/10^6^ seeded cells in the DC-S condition, 3.2 ± 0.26 U/day/10^6^ seeded cells in the DC-D condition, 3.58 ± 0.6 U/day/10^6^ seeded cells in the ECM-S condition, and 3.1 ± 0.21 U/day/10^6^ seeded cells in ECM-D the condition, probably related to HUVEC seeding. In all conditions, LDH release decreased from day 5 of the culture, indicating high cellular viability.

High cell activity was confirmed by the albumin production and CYP3A4 activity levels. Considering albumin, in the dynamic conditions, PHHs produced higher levels of albumin compared to static conditions, for both considered platforms [Fig. 4(e)(i)]. In particular, albumin levels were 2.03 ± 1.6, 8.89 ± 2.96, 11.31 ± 2.63, 6.47 ± 3.15 *μ*g/day/10^6^ seeded cells for the DC-S condition; 9.95 ± 3.65, 13.24 ± 3.01, 14.68 ± 3, 8.6 ± 0.87 *μ*g/day/10^6^ seeded cells for the DC-D condition; 2.26 ± 0.05, 6.64 ± 0.23, 24.29 ± 3.65, 15.57 ± 4.33 *μ*g/day/10^6^ seeded cells for the ECM-S condition; and 9.5 ± 4.1, 12.23 ± 3.79, 22.82 ± 3.08, 16.5 ± 1.49 *μ*g/day/10^6^ seeded cells for the ECM-D condition, on day 3, 5, 7, and 9, respectively. Albumin production trend was similar among all conditions, with the highest production on day 7 of the culture. Regardless of the culture condition, hepatic functions were significantly higher in the ECM device compared to the DC platform from day 7 of the culture. This may be due to the media transport regions difference between the two configurations, highlighting the importance of spatial gradients in organs-on-chips systems.

For what concerns CYP3A4 activity, the level was similar in all conditions, with no statistical differences [Fig. 4(e)(ii)]. In particular, CYP3A4 levels were 21.04 ± 3.18, 18.7 ± 2.97, 17.78 ± 1.58, and 19.21 ± 1.93 pmol/h/10^6^ seeded cells on day 7 for the DC-S, DC-D, ECM-S, and ECM-D conditions, respectively.

Overall, these results confirm the importance of replicating the space of Disse within a LoC model. In particular, albumin production in the ECM-D condition follows the trend of the DC-D model at the beginning of the culture, while static conditions remain lower, and then it increases on day 7 of the culture approaching values closer to the physiologic production.^4^ Similar to the hepatic monoculture, the co-culture of hepatocytes and endothelial cells in a physiologic-like environment allows to obtain higher CYP3A4 activity compared to the one of freshly isolated hepatocytes.^4^ However, the inclusion of the endothelial phenotype in the model does not seem to drastically improve CYP3A4 activity compared to when hepatocytes are cultured alone. It would be interesting to introduce in the model liver sinusoidal endothelial cells (LSECs) as a more representative endothelial cell type and test their effect on hepatic functionality.

### E. Molecular diffusion within the vascularized hepatic models

**FIG. 5.**
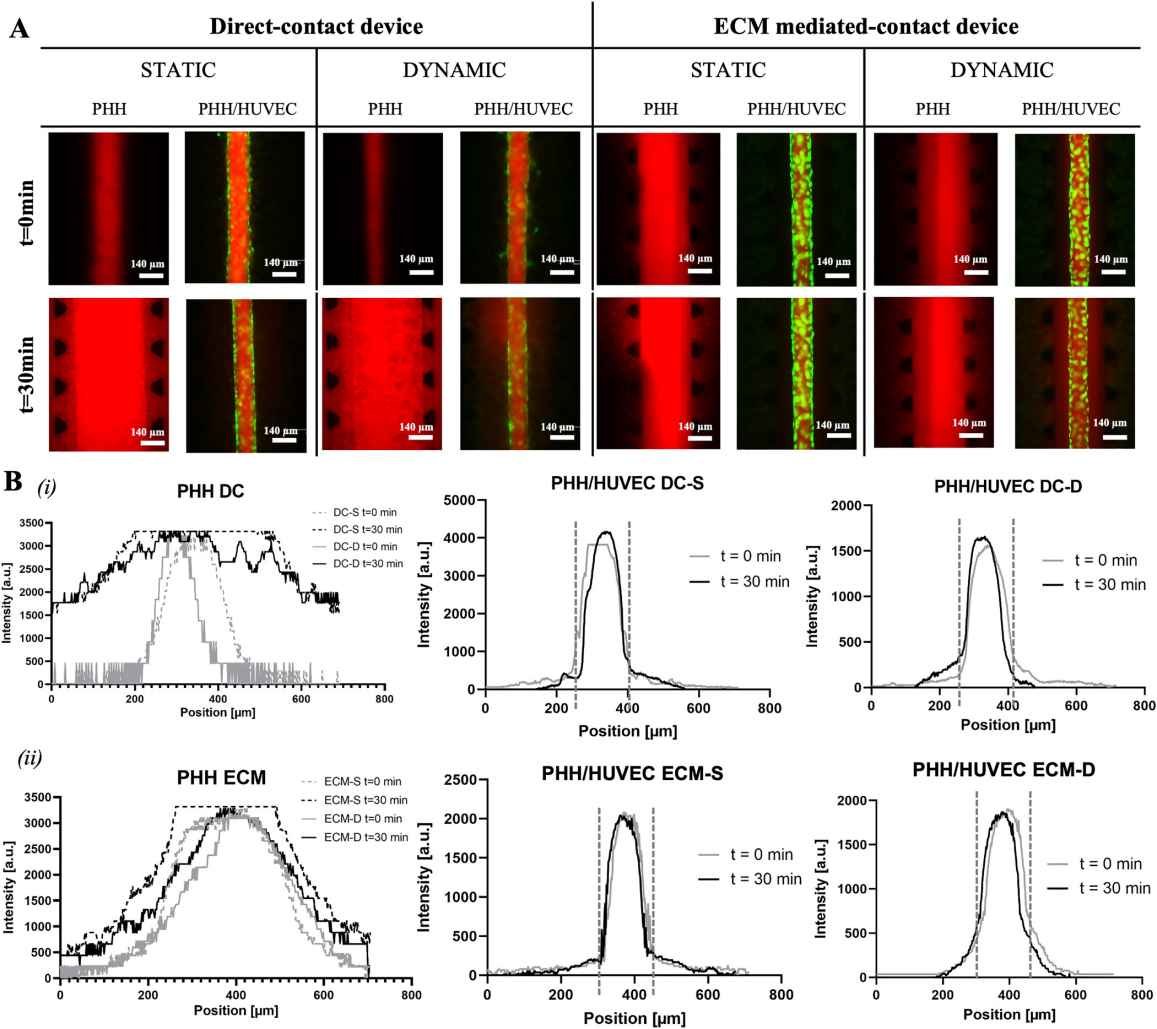
Permeability analysis within the liver sinusoid models generated in the devices after being exposed to static or dynamic culture conditions. (a) Images of the 40 kDa RFP-Dextran flowing within the lumen immediately after injection (t = 0) and after 30 min (t = 30). Scale bars are 140 *μ*m. (b) Cross-diameter bell-curve distribution of the fluorescent intensity immediately after RFP-Dextran injection and after 30 min in PHH-only (left) and in PHH/HUVEC (right) conditions within (i) direct-contact device (DC) and (ii) ECM-mediated-contact device (ECM), either cultured in static (-S) or dynamic conditions (-D).

To assess whether the bidirectional flow introduced by the rocking movement could impair a correct HUVEC channel generation, the permeability of the models was tested by perfusing 40 kDa RFP-Dextran on day 9 of the culture. Figure 5(a) shows that when the endothelial monolayer is not present in the lumen of the central channel, the 40 kDa Dextran immediately starts to diffuse in the whole device, whereas when the endothelial channel is formed, the diffusion of the fluorescein is limited owing to the ability of HUVEC junctions to retain the dye within the lumen. In particular, in both platforms, the endothelial channel appears tighter when the devices are in static conditions, as even after 30 min, the Dextran did not diffuse outside the lumen. Conversely, when the devices are placed on the rocker platform and the bidirectional flow is imposed, the endothelial lumen is able to retain the dye for only a few minutes, and after 30 min, the dye is already diffusing outside the lumen. These findings were quantified by calculating permeability coefficients from the bell-curve distribution of RFP-dextran fluorescent intensity [Fig. 5(b)], which resulted in higher than 1 × 10^−5^ for the PHH-only (both devices and conditions) and 1.9 × 10^−6^, 3 × 10^−6^, 1.2 × 10^−6^, and 5.4 × 10^−6^ cm/s for PHH/HUVEC DC-S, DC-D, ECM-S, and ECM-D, respectively. Thus, HUVECs are better organized in the static conditions for both devices as the permeability decreases in tighter endothelial monolayers. However, the native liver is populated by the liver sinusoidal endothelial cells (LSECs) that contain numerous fenestrations acting as a selective filtration system in the order of 50–300 nm,^21,22^ i.e., in the dimensional range of the dye used in our study. Culture conditions with bidirectional flow could, thus, make HUVEC a candidate cell source to recapitulate the presence of the native fenestrae.

## III. CONCLUSIONS

In this paper, we showed the development of an efficient method to generate perfusable endothelial channels within 3D microtissues without interposing membranes or scaffolds. Thanks to the proposed technique, we successfully designed, fabricated, and validated two liver-on-chip models, aiming to mimic the architecture of the liver sinusoid *in vitro*. The direct-contact platform allows for direct endothelial–hepatic communication, whereas in the ECM-mediated-contact platform, a 100 *μ*m-thick collagen–fibrin layer separates the endothelial channel from the 3D hepatic construct, better resembling the presence of the space of Disse located in the liver sinusoid *in vivo*. In comparison to other microfluidic studies,^8^ the collagen–fibrin hydrogel adopted in our study displays a stiffness similar to the *in vivo* liver matrix.^15,16^ We evaluated hepatic functions by means of albumin production and CYP3A4 activity, investigating two culture conditions (i.e., static vs bidirectional flow). In both devices, under dynamic conditions, hepatocytes expressed high albumin and CYP3A4 levels in agreement with previous studies.^7–9,23^ Although being able to recapitulate the presence of the endothelial fenestrations, the effect of the bidirectional flow on endothelial cell alignment needs to be further evaluated. An interesting hint could be the introduction of a unidirectional flow in the central channel of the platforms to assess whether a different fluid motion could be beneficial in generating more aligned endothelial channels and see a reflection on hepatic functionality.

Nonetheless, the devices here developed in the context of the vascularized liver are in truth versatile and can be used for various purposes. The proposed platform could be exploited to mimic different organ models where low seeding volumes are needed and modeling vasculature is pivotal, such as the blood–brain–barrier and tumor microenvironment. Moreover, this methodology paves the way to numerous further uses of microfluidic technologies coupled with vascularized tissue models, for instance, the introduction of the immune system perfusion within the vasculature channel and the integration within multiorgan-on-chip platforms to study communication among vascularized organs (i.e., a slightly modified version of the device toward multiorgan-on-chip integration is presented in supplementary material S.4).

## IV. EXPERIMENTAL SECTION

### A. Microdevices design and fabrication

The layouts of the microfluidic devices [i.e., Direct-contact (DC) and ECM-mediated-contact (ECM)] were generated through specific computer-aided (CAD) software (AutoCAD^®^, AutoDesk Inc., USA). The complete structure of the devices was conceived through the design of two different functional layers: (i) a microfluidic channel layer for cell culture and (ii) a tank layer for cell culture. Features heights were set as follows: 200 *μ*m for the channel layer and 50 *μ*m for the tank layer. Silicon master molds were fabricated in a cleanroom environment (Polifab, Politecnico di Milano) through SU-8 (SU8-2050 and SU8-2100, MicroChem, USA) photolithography. Briefly, we first adopted flood exposure to increase adherence of SU-8 features to the silicon wafer, and then the pattern of each layer was transferred onto either SU8-2050 or SU8-2100 photoresists, previously spin-coated (Spin Coater Karl Süss RC8, Süss Microtec, Germany) on 4 in. silicon wafers. Specifically, to obtain 200 *μ*m height of the culture layer, two subsequent SU8 exposures were performed: first, a SU8-2100 layer of 150 *μ*m was spin-coated on the silicon wafer with a spinning velocity of 2800 rpm and then a SU8-2050 layer of 50 *μ*m was spin-coated on the silicon wafer with a spinning velocity of 3500 rpm so to increase the height of the central culture channel only. Concerning the design of the DC platform, the microfluidic channel layer was composed of one central channel (520 *μ*m wide) separated from two flanking media channels (1 mm wide) by two arrays of trapezoidal microposts (56 *μ*m smaller base and 160 *μ*m larger base). Additionally, the channel layer displays one self-sealing micro-guide of squared cross section (side of 150 *μ*m) that was designed with a widening of about 1 mm at the outer border to facilitate the manual insertion of a micro-needle. The micro-guide was separated from the central channel by a 200 *μ*m pierceable and self-sealing wall.^13^ Moreover, the central culture channel contains a couple of inner (1.5 mm diameter) and outer (5 mm diameter) reservoirs, for cell-laden hydrogel injection and endothelial cell seeding, respectively. The 1.5 mm reservoir is connected to the 5 mm one by a diverging channel, where the smaller width is 120 *μ*m (in contact with the small reservoir) and the larger width is 300 *μ*m (in contact with the big reservoir). Regarding the fabrication of the tank layer, SU8-2050 was spin-coated on the silicon wafer with a spinning velocity of 3500 rpm to obtain 50 *μ*m height. Additionally, the tank layer is composed of a unique 450 *μ*m wide central channel that needs to be aligned to the central channel. Concerning the design of the ECM platform, the microfluidic channel layer was composed of one central channel (320 *μ*m wide), two adjacent culture channels of 300 *μ*m width each, again separated from two flanking media channels 1 mm wide. Every channel is separated from the adjacent one by two arrays of trapezoidal microposts. Additionally, the channel layer displays one self-sealing micro-guide of squared cross section designed as previously described. Regarding the fabrication of the tank layer, SU8-2050 was spin-coated on the silicon wafer with a spinning velocity of 3500 rpm to obtain 50 *μ*m height. Additionally, the tank layer is composed of a unique 250 *μ*m wide central channel that needs to be aligned to the central channel of the microfluidic culture layer. All layers were fabricated by direct laser writing (Heidelberg MLA100, Heidelberg Instruments). The channel layer is placed above the tank layer (inverted), so that the central channel is precisely aligned on top of the tank to generate a total height of the central channel of 250 *μ*m.

The microstructured silicon master molds were used for the soft-lithographic process. First, the master molds were subjected to a silanization treatment. Briefly, the mold surface is exposed to tri-methyl-chloro-silane (TMCS, Sigma-Aldrich) for 30 min at room temperature to favor PDMS removal and preserve the integrity of the silicon mold. PDMS layers were thus fabricated through replica-molding of PDMS (Sylgard 184, Dow Corning; mixing ratio of 10:1 elastomer base: curing agent) on master molds. After curing (65 °C for 2 h), PDMS layers were peeled off the mold and assembled by aligning the channel layer to its correspondent tank layer by air plasma (Harrick Plasma Inc). A 1.5 mm diameter biopsy puncher was used to punch inner inlets/outlets of the cell culture chambers in the channel layer, 5 mm diameter biopsy puncher was used to punch inlet/outlet of the outer cell culture chambers creating medium reservoirs for the internal luminal culture in the channel layer and 6 mm diameter biopsy puncher was used to punch inlets/outlets of the flanking media channels in the channel layer to generate medium reservoirs. Additionally, 5 mm diameter biopsy puncher was used to open the outer border of the self-sealing micro-guide to allow needle insertion/removal.

### B. Technical characterization of the lumen

Before biological experiments, devices were first characterized by means of bead perfusion within the central channel. To obtain a cylindrical lumen within the central channel, a 30% fibrin (2.1 mg/ml, human fibrin, Sigma)—70% collagen (9 mg/ml, rat tail collagen I, Corning) gel without cells was injected either in the central channel of the DC platform or the central- and midchannels of the ECM platform, following the steps previously described. To verify lumen formation, polystyrene microbeads were injected through the outer seeding ports of the central lumen after needle removal.

### C. Cell culture and seeding procedure

HUVEC and primary human hepatocytes (PHH) were adopted as endothelial and hepatic cell types, respectively. GFP-HUVECs were purchased at P0 (Lonza, Switzerland). Cells were expanded from P0 to P1 and then stored in liquid nitrogen and used from P1 to P4. HUVECs were cultured in 75 cm^2^ flasks pre-coated with fibronectin (Sigma-Aldrich, St. Louis, USA, 10 *μ*g/ml) in the Vasculife basal medium supplemented with Vasculife VEGF LifeFactor kit containing 2% (v/v) of fetal bovine serum (FBS), 1 *μ*g/ml of hydrocortisone, 5 ng/ml of human Fibroblastic Growth Factor basic (hFGF-B), 5 ng/ml of Vascular-Endothelial Growth Factor (VEGF), 15 ng/ml of Recombinant human Long Insulin Like Growth Factor-1 (R3-IGF-1), 50 *μ*g/ml of ascorbic acid, 5 ng/ml of human Epidermal Growth Factor (hEGF), 30 *μ*g/ml and 15 ng/ml of Gentamicin sulfate and Amphotericin-1000 (GA-1000), 10 mM of l-glutamine, and 0.75 U/ml of heparin sulfate hereafter referred to as endothelial medium. Cells were maintained at 37 °C, 5% CO_2_, and the medium was changed every 48 h until 80% confluency was reached. Prior to seeding, the medium is removed from the flask, and cells are washed with 10 ml of PBS ([−]CaCl_2_ [−]MgCl_2_). Then, 3 ml of Trypsin-EDTA (EuroClone, Milan, Italy) was added for 4 min inside the incubator (37 °C, 5% CO_2_). Cellular detachment was checked by controlling the floating cells under the microscope, and then 7 ml of the medium were added to inhibit trypsin. The cellular suspension was transferred into a 50 ml falcon tube and 10 *μ*l harvested for counting, while the cells in the falcon were centrifuged for 5 min at 300 × g. The supernatant was discarded and the cell pellet at the bottom resuspended in an appropriate quantity of endothelial medium at 20 M/ml concentration. Cryopreserved PHH (donor Hu2098, Thermo Fisher Scientific) were thawed in the PHH seeding medium consisting of William's E (Thermo Fisher Scientific, no glucose) supplemented with 15 mM HEPES (Thermo Fisher Scientific), 2 mM Glutamax (Thermo Fisher Scientific), 5.5 mM glucose, 200 pM insulin, 100 nM hydrocortisone, 5% FBS, and 1% PEN/STREP; centrifuged at 50 × g for 8 min; and seeded at 20 M/ml or 40 M/ml in the fibrin–collagen gel. Collagen and fibronectin are the main structural constituents of the liver ECM,^16,24^ and fibrin has innate angiogenic properties.^25^ One day after seeding, the PHH seeding medium was replaced with PHH maintenance medium consisting of William's E (Thermo Fisher Scientific, no glucose) supplemented with 15 mM HEPES (Thermo Fisher Scientific), 2 mM Glutamax (Thermo Fisher Scientific), 5.5 mM glucose, 200 pM insulin, 100 nM hydrocortisone, 2% FBS, 0.5% PEN/STREP, 1.25 mg/ml fatty-acid free BSA, 6.25 ng/ml sodium selenite, 6.25 *μ*g/ml transferrin, and 20 *μ*M linoleic acid.

The seeding procedure is highlighted in Figs. 1(b) and 4(b). Briefly, a 120 *μ*m-diameter needle is inserted through the guide of the devices, and then hepatocytes (20 or 40 M/ml) are seeded in 30% fibrin (Sigma)–70% collagen (Corning) gel at 9 mg/ml and 2.1 mg/ml final concentration in the inner seeding port of the direct-contact platform and in the two midseeding ports of the ECM-mediated-contact platform. Briefly, rat tail collagen I (4.39 mg/ml stock) was mixed with 1/10 PBS 10×, NaOH (2% of collagen stock), and PBS 1× to reach a final concentration of 3 mg/ml. We checked the pH of the obtained collagen gel and validated its suitability for cell culture (i.e., pH 7.4). Thrombin (100 U/ml stock) was mixed with the PHH seeding medium in a ratio of 1:9 to reach a final concentration of 10 U/ml; fibrinogen (100 mg/ml stock) was mixed with PBS 1× in a ratio of 3:2 to reach a final concentration of 60 mg/ml. 10 *μ*l fibrin–collagen solution was prepared by mixing 7 *μ*l collagen, 1.5 *μ*l thrombin, and 1.5 *μ*l fibrinogen solutions and injected in the central channel of the DC or the two midchannels of the ECM platforms. After 15 min in the incubator medium, it is inserted in the lateral media channels of the devices. At this point, in the ECM-mediated-contact platform, a 30% fibrin–70% collagen gel (10 mg/ml and 2.1 mg/ml final concentration) without cells is injected in the central inner seeding port to surround the needle. The diverging channels previously described are, thus, needed to precisely confine the hydrogel within the inner section of the central channel, avoiding gel leakage toward the outer reservoirs. 15 min after hydrogel reticulation in the incubator, the needle is removed, and the PHH seeding medium is added in the outer seeding ports of the central channel to hydrate the lumen. On day 2 of the culture, 10 *μ*g/ml fibronectin (Sigma, 1 mg/ml) solution in PBS is injected through the outer seeding ports of the central channel and after 1 h, fibronectin is removed, and the lumen is rinsed with endothelial medium before HUVEC seeding. HUVECs at 20 M/ml were deposited in the outer port (3 *μ*l being the best working volume tested) of the central channel near the channel opening, with a 10 *μ*L pipet. Cells were allowed to flow toward the outer port for a few minutes, thanks to the difference in pressure, until a pressure equilibrium was reached. When the channel was filled with cells, the platforms were incubated upside-down (around the longitudinal axis) using a humidified box, for 30 min inside an incubator (37 °C, 5% CO_2_) to guarantee a uniform distribution of cells. This passage was repeated with the devices in normal position until cells homogeneous attachment to the whole lumen length (∼5 mm) was achieved. Then, a culture medium composed of 50% PHH maintenance medium and 50% endothelial medium was added to the culture and changed every 24 h. Devices were either cultured in static conditions or placed on a rocker platform (VWR, 15° tilting angle, 1 rpm velocity, see supplementary material S.3) from day 0 to day 9 of the culture. This dynamic movement induces a shear stress of 0.9 dyne/cm^2^ within the central endothelial lumen according to Eq. (1), which is in line with the shear stress experienced by LSECs in the liver sinusoid,^9^

$$\tau =\frac{r}{2}\rho g\;\sin(\alpha ),$$ (1)

where τ is the shear stress generated in the lumen, *r* is the radius of the lumen (i.e., ∼70 *μ*m), ρ is the density of the medium (i.e., water, 1000 kg/m^2^), *g* is the gravitational force (i.e., 9.81 m/s^2^), and α is the tilting angle (i.e., 15°).

### D. Viability and functionality assessment

To optimize the model from the biological point of view, we first studied the formation of the internal endothelial channel using HUVECs alone. Meanwhile, we analyzed the hepatic behavior in the 3D fibrin–collagen gel within the DC device by means of PHH cells, and finally, we assessed the functionality of the liver sinusoid models when HUVECs and PHHs are co-cultured together in both platforms.

#### 1. Lumen characterization with HUVECs

HUVEC morphology was assessed and controlled every other day using the EVOS FL microscope (Thermo Fisher Scientific) with 4×, 10×, and 20× phase contrast objectives. All acquisitions during the optimization of the endothelial cells seeding were performed with LeicaSP8 AOBS confocal microscope at 10× or 25× magnification (10×/0.3 dry, 25×/0.95 water), for bright field (BF) and fluorescent acquisition, respectively. Tile scan single plane BF, confocal z-stack, and y-stack images of the entire length of the 3D-chip channel were acquired using the LasX navigator, merged by LASX software, and rendered in 3D with Leica LasX 3D viewer.

#### 2. Model optimization and characterization with PHH

First, the direct-contact device was used to optimize the seeding density condition and culture medium composition. In particular, we tested medium effects when it was supplemented with 2 *μ*g/ml ACA on 20 and 40 M/ml PHH. The morphology of the cells was assessed and controlled every other day using the EVOS FL microscope (Thermo Fisher Scientific) with 4×, 10×, and 20× phase contrast objectives. Cell viability was monitored for the whole culture time by analyzing the production of lactate dehydrogenase (LDH) or through a live/dead assay. Quantification of LDH is a well-established assay for cell viability, as when a tissue is damaged, LDH is released from the cytoplasm into the extracellular environment.^26^ To assess LDH release from the culture, culture supernatants were collected every 2 days for up to day 9. Briefly, LDH concentration was assessed using the LDH-Glo™ Cytotoxicity Assay (Promega) following manufacturer instructions. To measure luminescence, SpectraMax^®^ i3x (Molecular Devices) was adopted. Cell viability was also assessed through live/dead (diluted in 1× PBS, Thermo Fisher Scientific) immunofluorescent staining. In detail, PHH was stained for 15 min in the incubator and fluorescent images were acquired and analyzed by the EVOS FL microscope (Thermo Fisher Scientific) with 4×, 10×, and 20× phase contrast objectives. For what concerns hepatic functionality, culture supernatants were collected every 2 days for up to 9 days and assessed for albumin concentration. Briefly, human albumin concentration was assessed using a competitive enzyme-linked immunosorbent assay (ELISA, DuoSet, R&D Systems) with horseradish peroxidase detection and the substrate 3,3′,5,5′-tetramethylbenzidine (TMB, Rockland Immunochemicals, Boyertown, PA). The cytochrome P450 enzyme family (CYP450) in the liver mediates the phases of drugs and xenobiotic metabolism. Of the CYP450 family, the enzyme CYP3A4 is present in the highest quantity and participates in the biotransformation of over 50% of commonly used drugs. To measure CYP3A4 activity, on day 7 of the culture, devices were incubated with the PHH maintenance medium supplemented with Luciferin-IPA (Promega, 1:1000 dilution) for 1 h in the incubator. The CYP medium was collected and devices with cells were then washed with the PHH maintenance medium. The CYP medium was addressed for CYP3A4 activity by means of the P450-Glo™ CYP3A4 luminescence assay by means of the standard curve generated with the given luciferin standards. Specifically, to measure absorbance and luminescence, SpectraMax^®^ i3x (Molecular Devices) was used.

### E. Diffusion test

The permeability of the models was tested by means of the perfusion of a 40 kDa RFP-Dextran on day 9 of the culture. Specifically, 40 kDa RFP-Dextran (Sigma) was diluted in PBS to reach 1 mg/ml concentration, and 50 *μ*l fluorescein solution was injected in one of the outer ports of the central culture channel of either the direct-contact and ECM-mediated-contact platforms. Images at 0-, 10-, 20-, and 30-min time points were acquired with the fluorescent microscope EVOS FL (Thermo Fisher Scientific) with 10× objective. The permeability was calculated considering only a central region of interest (ROI) encompassing the culture channels. Intensity profiles were derived using ImageJ software. The permeability (cm/s) was calculated using Eq. (2),^11^

$$P=\frac{r\;dI}{2\;\Delta Io\;dt\;}$$ (2)

where *r* is the lumen radius, Δ*Io* is the initial increase in fluorescent intensity right after Dextran injection, and *dI/dt* is the rate of increase in fluorescence intensity as Dextran exits into the gel.

## SUPPLEMENTARY MATERIAL

Further data on collagen–fibrin gel characterization, immunofluorescent staining of peculiar endothelial markers, rocker platform setup, and an upgraded version of the current ECM-mediated-contact platform are presented in the supplementary material.

## Data Availability

The data that support the findings of this study are available from the corresponding author upon reasonable request.
